# The current use of feasibility studies in the assessment of feasibility for stepped-wedge cluster randomised trials: a systematic review

**DOI:** 10.1186/s12874-019-0658-3

**Published:** 2019-01-10

**Authors:** Caroline A. Kristunas, Karla Hemming, Helen Eborall, Sandra Eldridge, Laura J. Gray

**Affiliations:** 1 Department of Health Sciences, University of Leicester, Leicester, UK; 2 Institute of Applied Health Research, University of Birmingham, Birmingham, UK; 3 Centre for Primary Care and Public Health, Queen Mary University of London, London, UK

**Keywords:** Stepped-wedge trial, Cluster randomised trial, Pilot trial, Feasibility study, Systematic review

## Abstract

**Background:**

Stepped-wedge cluster randomised trials (SW-CRTs) are a pragmatic trial design, providing an unprecedented opportunity to increase the robustness of evidence underpinning implementation and quality improvement interventions. Given the complexity of the SW-CRT, the likelihood of trials not delivering on their objectives will be mitigated if a feasibility study precedes the definitive trial. It is not currently known if feasibility studies are being conducted for SW-CRTs nor what the objectives of these studies are.

**Methods:**

Searches were conducted of several databases to identify published feasibility studies which were designed to inform a future SW-CRT. For each eligible study, data were extracted on the characteristics of and rationale for the feasibility study; the process for determining progression to the main trial; how the feasibility study informed the main trial; and whether the main trial went ahead. A narrative synthesis and descriptive analysis are presented.

**Results:**

Eleven feasibility studies were identified, which included eight completed study reports and three protocols. Three studies used a stepped-wedge design and these were the only studies to be randomised. Studies were predominantly of a mixed-methods design. Only one study assessed specific features related to the feasibility of using a SW-CRT and one investigated the time taken to complete the study procedures. The other studies were mostly assessing the feasibility and acceptability of the intervention.

**Conclusion:**

Published feasibility studies for SW-CRTs are scarce and those that are being reported do not investigate issues specific to the complexities of the trial design. When conducting feasibility studies in advance of a definitive SW-CRT, researchers should consider assessing the feasibility of study procedures, particularly those specific to the SW-CRT design, and ensure that the findings are published for the benefit of other researchers.

**Electronic supplementary material:**

The online version of this article (10.1186/s12874-019-0658-3) contains supplementary material, which is available to authorized users.

## Background

The stepped-wedge cluster randomised trial (SW-CRT) is a novel and appealing trial design which can be used for the evaluation of interventions during routine implementation [1, 2]. The design involves randomisation of clusters to sequences which dictate the order at which the clusters cross from the control to the intervention condition [3]. In general, this involves all clusters starting the trial in the control condition, followed by a staggered introduction of the clusters to the intervention, resulting in all clusters receiving the intervention by the end of the trial [3]. The implementation of interventions under evaluation can often proceed in much the same way as it would have had the evaluation not been taking place, with the exception of the order of implementation [3]. This means the design has the potential to provide real world evidence of effectiveness; that can be generalised; and can be implemented with minimal disruption. With the increasing availability of routinely collected data, the trial design has the potential to become the gold standard for the evaluation of implementation and quality improvement interventions.

The use of the SW-CRT is seeing an unprecedented and exponential increase [4]. However, some of the complexities of the trial design can put studies at risk of not delivering on their objectives [5, 6]. In particular, due to the staggered implementation of the intervention, there is the potential for the scheduled timings of the trial to be disrupted. Disruptions to the planned implementation schedule and organisation of the trial may have repercussions that ultimately result in the trial being unsuccessful. In addition, it is generally required for all clusters to be recruited prior to any randomisation taking place. If recruitment of clusters is slower than expected then this can severely delay the start of the trial or could result in fewer clusters being recruited and an underpowered trial. Getting the timings of the trial right, particularly the timing of the clusters starting the intervention can be a challenge. Without first testing the implementation of the intervention, it may be difficult to determine how long the intervention will take to embed in a cluster, and therefore how long the periods need to be between clusters starting the intervention. Maintaining consistency in participant recruitment over the duration of the trial may be difficult, especially when the cluster changes from control to intervention condition. When continuously recruiting throughout the trial variations in the number or type of participants can occur. These variations can relate to variations in the level of engagement from those recruiting participants, which may wane as the trial progresses, or as a result of staff turnover. These challenges surrounding the design and conduct of SW-CRTs mean feasibility studies could be particularly useful.

Feasibility and pilot studies are small scale studies conducted prior to a definitive trial. They aim to guide the planning or design of the trial, or determine whether the main trial is feasible and if not, what issues, if any, can be resolved to make the main trial feasible [7–10]. Feasibility studies can be designed to investigate a vast range of issues [11]. Some may be focussed on testing the feasibility of the intervention; for example whether the intervention is acceptable to its intended recipients; whether it is suitable for the environment where it will be introduced; and whether there are any challenges that might arise during the implementation [11, 12]. Other feasibility studies may be more concerned with assessing the feasibility of the trial processes: for example testing the methods of data collection; the acceptability of the randomisation or recruitment procedures; or testing if there are sufficient resources available to conduct the trial [12]. Depending on its objectives a feasibility study may or may not have the same design as the main trial. Pilot studies can be defined as a subset of feasibility studies, where pilot studies have a particular design feature [10, 13]. A pilot study is conducted as a small scale version of all or part of the future definitive RCT (that may or may not be randomised) [10, 13]. Throughout, the term feasibility study will be used to encompass both feasibility and pilot studies.

Testing and refining the trial processes for SW-CRTs will be pivotal to their success. For example, it may transpire that due to resource availability or the complexity of the intervention, a limited number of clusters can simultaneously cross from the control to the intervention. The resource levels needed to start and maintain a cluster in the intervention condition can be investigated in a feasibility study. The length of time required between clusters crossing to the intervention might also be important; and again can be investigated in a feasibility study. This time should be long enough to allow the intervention to become embedded in a cluster before a measure of the outcome is obtained, whilst being short enough to allow the trial to complete within a set funding period. It might also be important to determine if recruitment of participants can be done in such as way so as not to be influenced by any knowledge of the intervention condition (which can induce biases) [14].

Up until now, it has not been known whether feasibility studies are being used to inform the design of SW-CRTs and if they are, which issues are being investigated. A recent systematic review of SW-CRTs [4] identified three pilot studies for SW-CRTs, which were themselves of a stepped-wedge design. However, since not all published feasibility studies for SW-CRTs will have a stepped-wedge design, not all will have been identified by previous reviews. This review aims to gain an insight into how feasibility studies are being used to inform the design of SW-CRTs. Specifically, our objectives were to:

- Systematically identify published feasibility studies designed to inform SW-CRTs;
- Ascertain the design characteristics of and rationale for these feasibility studies;
- Establish how the feasibility studies informed the main trials.

## Methods

### Search strategy

Feasibility studies for SW-CRTs, published in English, were identified via electronic searches conducted on 6th February 2017 of the online published databases Ovid MEDLINE (from 1946), Scopus (from 1966), Embase (from 1947) and PsycINFO (from 1967). An example of the search strategy used is outlined in Table 1 [15] and was based on previously published search strategies [1, 7, 12, 16, 17].

**Table 1:** Example search strategy for Ovid MEDLINE

1. “pilot*”.mp 2. “feasibil*”.mp 3. 1 OR 2 4. “step* wedge*”.mp 5. “step*wedge*”.mp 6. “delay* intervention”.mp 7. “experimental* staged introduction”.mp 8. (“one* direction* crossover design” OR “one* direction* cross* over design”).mp 9. (“incremental* recruitment” OR “incremental* introduction” OR “incremental* implementation” OR “incremental* allocation”).mp 10. (“phased* recruitment” OR “phased* introduction” OR “phased* implementation” OR “phased* allocation”).mp 11. (“staggered* recruitment” OR “staggered* introduction” OR “staggered* implementation” OR “staggered*allocation”).mp 12. (“stepwise* recruitment” OR “stepwise* introduction” OR “stepwise* implementation” OR “stepwise*allocation”).mp 13. (“step*wise* recruitment” OR “step*wise* introduction” OR “step*wise* implementation” OR “step*wise*allocation”).mp 14. (“delayed* recruitment” OR “delayed* introduction” OR “delayed* implementation” OR “delayed*allocation”).mp 15. or/4–14 16. 3 AND 15 17. limit 16 to English language	

* truncation symbol, for example "pilot*" retrieves "pilots" as well as "pilot"

### Inclusion criteria

Eligible studies were full reports or protocols of feasibility studies conducted to inform a future SW-CRT. For the purpose of this review, a feasibility study for a SW-CRT was defined as any study which aimed to ascertain the feasibility of a planned SW-CRT, through the assessment of issues other than solely the refinement of the intervention. We consider pilot studies to be a subset of feasibility studies and were therefore eligible for inclusion in this review.

No restrictions were placed on the design of the feasibility study, so it was not necessary for the feasibility study to be of a stepped-wedge design, or even randomised. The feasibility study should, however, have focussed objectives to ascertain the feasibility of a planned SW-CRT and make it clear how the findings of the study will inform the main trial, which must be intended to be of an SW-CRT design. An SW-CRT is defined as any trial that randomises clusters to two or more steps (time-points at which clusters have a unidirectional change of treatment condition). Studies for which the intended definitive trial is individually randomised, has a bidirectional cross-over design or is non-randomised have been excluded.

### Screening

Two reviewers (CK & Maria Yao) independently and in a random order screened the titles and abstracts of the identified studies for eligibility. For those studies not excluded at the initial screening, full-text articles were obtained and the same duplicate method of assessment used. Ineligible studies were excluded and the reason for exclusion noted. If any additional information was required the authors were contacted and attempts were made to access any protocols for the identified feasibility studies. For each eligible study, the reference lists were also checked for any potentially eligible studies.

### Data extraction

Data extraction for eligible studies was done independently and in duplicate in a random order by two reviewers (CK & (KH or LG)), using a data extraction form that had been tested, revised and finalised using a small number of the studies. Extracted data was managed in Microsoft Excel V.2013.

Information about the design of each feasibility study was extracted. This included how the authors defined their study (as a pilot, feasibility study or something else); the size of the study and how the sample size was justified; and whether the study has been registered with a recognised clinical trial registry, such as ClinicalTrials.gov. In addition, information was extracted on blinding, randomisation and overall design of the feasibility study (parallel, stepped-wedge etc.). The rationales for conducting each feasibility study prior to a main trial were obtained by extracting the specific aims of each study. These were categorised into process (feasibility of the processes that take place during the trial), resource (time, people and budget issues), management (feasibility of collaborations and coordination of teams) and scientific type (assess scientific processes and estimation of parameters) motivations (more detail is included in the published protocol [15]).

Information on the types of analysis conducted and the emphasis put on any results were extracted. Information was also extracted on any hard or soft stopping rules that were in place and the criteria used to determine whether the main trial would be feasible or not. Whether the decision was made to go ahead with the main trial and how the feasibility study informed or resulted in changes being made to the main trial were recorded, along with any information on whether any of the participants from the feasibility study would also be taking part in the main trial.

### Analysis of results

We present a narrative synthesis of our findings, as well as a descriptive analysis of the study characteristics of each eligible feasibility study included in the review. We also present a critical appraisal of a single case study which highlights specific issues regarding feasibility studies and SW-CRTs [Additional File 1]. Where appropriate, this report adheres to the Preferred Reporting Items for Systematic reviews and Meta-Analyses (PRISMA) statement [18].

## Results

**Fig. 1:**
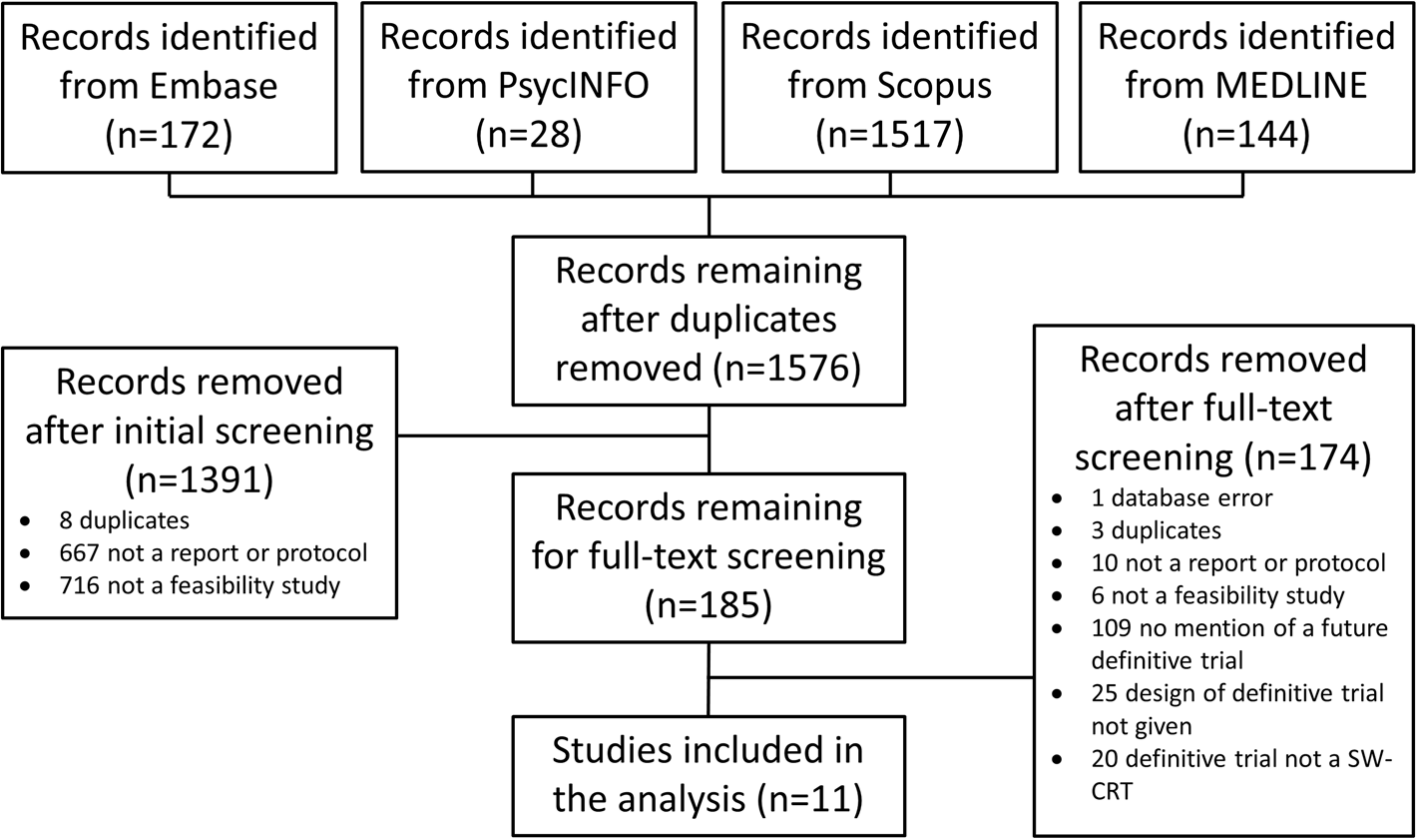
Flow diagram: The flow of information through the different stages of the systematic review

A total of 1861 records were identified from the search of the databases, of which 11 studies were found to be eligible and are included in this analysis [Fig. 1; Additional File 2].

Of these 11 studies included, less than half reported that the study had been registered (Table 2) [Additional File 3]. The majority of the identified studies were reports of results, but three protocols were also identified. Just over half of the studies described themselves as a pilot study, with the others using terms such as “feasibility study”, “acceptability and feasibility pilot”, “consultation exercise” or “formative research”.

**Table 2:** Characteristics of identified feasibility studies

Characteristic	Number (%)^a^
Study registered	5 (45)
Report of findings	8 (73)
Protocol	3 (27)
Study described as:	
Pilot	6 (55)
Other	4 (36)
Not described	1 (9)
External feasibility study	8 (73)
Internal feasibility study	2 (18)
Unclear	1 (9)
Type of research:	
Quantitative	3 (27)
Qualitative	1 (9)
Mixed	6 (55)
Unclear	1 (9)
Quantitative design:	
Single-arm	4 (36)
Stepped-wedge	3 (27)
Parallel	1 (9)
Observational	1 (9)
Randomised	3 (27)
Setting:	
Health care	9 (82)
Community	2 (18)
Cluster type:	
Hospitals	3 (27)
Wards	4 (36)
Clinics	2 (18)
Geographical areas	2 (18)
Study duration (months) (median (IQR))	12 (4.7, 15)
Number of clusters (median (IQR))	3.5 (1.8, 6)
Participant type:	
Healthcare professionals	1 (9)
Patients	5 (45)
Both healthcare professionals and patients	4 (36)
Other	1 (9)
Number of participants (median (IQR))	109 (35, 2220)
Rational for sample size:	
Convenience sample	6 (55)
Calculation based on the main clinical outcome	2 (18)
Not stated	3 (27)

^a^with denominator the total number of studies (*n* = 11) unless otherwise stated, *IQR* interquartile range

Three of the identified studies were of a stepped-wedge design, with two of these having a qualitative component. Over half of the identified studies used mixed methods; and most (73%) were external to the intended main trial. The duration of the studies ranged from 16 weeks to two years, with a median duration of one year (interquartile range (IQR) (months): 4.7, 15). Three of the studies were randomised (all at the cluster level and all having a stepped-wedge design) with no restrictions to the randomisation mentioned. Blinding was present in one of these [19].

The majority of the included studies were conducted in health care settings, where the clusters were mostly defined as hospitals, clinics, or wards. The median number of clusters in each study was 3.5 (IQR: 1.8, 6). The study participants were mostly patients, healthcare professionals or both, including on average 109 (IQR: 35, 2180) participants. The majority of studies used a convenience sample and for three studies the rationale for the sample size was unclear.

### Rationales for conducting the feasibility study

The studies reported a range of rationales, including process and resource rationales, though none reported any management rationales [Table 3, Additional File 4].

**Table 3:** Rationales given for conducting the identified feasibility studies

Rationales given	Number
Process type motivations (n = 11):	
Acceptability of intervention	7
Identify issues/barriers to implementation	6
Adherence to intervention	4
Development of intervention	3
Retention rate estimation	3
Determining outcome measures	2
Test data collection methods	2
Assess amount of missing data	2
Other^a^	5
Resource type motivations (*n* = 3):	
Resources used in intervention	1
Post-intervention impact on service use and staff time	1
Time taken to complete study procedures	1
Waiting and consultation times	1
Patient volumes and staffing levels during study	1
Management type motivations (*n* = 0):	
None reported	11
Scientific type motivations (*n* = 8):	
Potential effectiveness of intervention	5
Assess cost-effectiveness/theoretical cost saving	3
Inform sample size calculation	3
Assess intervention safety	2
Estimate Intra Cluster Correlation Coefficient (ICC)	1
Assess correlation between measures	1
Assess distributional properties of measures	1

^a^participant satisfaction; assess values-treatment concordance; acceptability relevance and importance of trial; test sampling methodologies; test feasibility of using stepped-wedge design

n = the number of studies that gave any motivation of this type

The most common process type motivations were investigating acceptability of the intervention (64%); identifying issues or barriers to implementation (55%) and intervention adherence (36%). Six studies investigated outcome measure related process type motivations; either choice of outcome measure; testing of data collection methods; or assessing the amount of missing data. Only one study assessed the feasibility of the processes specifically relating to the use of the stepped-wedge design. A description of this study is given in [Additional File 1].

Only three of the identified studies stated any resource type motivations for conducting the study. Each of the following were assessed by one of the studies: the resources used in the intervention; the post-intervention impacts on service use and staff time; the time taken to complete study procedures; waiting and consultation times; and patient volumes and staffing levels. None of the feasibility studies identified listed any management type issues that they investigated.

Some scientific type motivations were investigated, the most common being an estimation of the potential effectiveness of the intervention (45%). In addition, some studies aimed to assess the cost-effectiveness/theoretical cost savings of introducing the intervention or intended to use information gained from the feasibility study to inform the sample size calculation.

### Progression to a main trial

Only two (18%) studies gave any criteria for determining the success of the feasibility study and deciding whether to proceed to a main trial. One study provided specific criteria, stating the threshold enrolment rate required and the lowest proportion of visits that needed to be completed and sessions that needed to be attended [20]. The criteria for the other study were not as specific: the completion of the economic modelling of the pilot data would “underpin the decision to progress to a main trial” [21].

One study put in place a stopping rule. The decision as to whether to continue the study was based on the change seen in the outcome from a period prior to the implementation of the intervention [21]. This study is described in more detail in [Additional File 1]. None of the feasibility studies that had been conducted at the time of this review were stopped prior to completion.

### Analysis method

The majority of the studies (55%) used a mixed-methods approach to analysing their data. The quantitative methods used included descriptive statistics, simple statistical tests and generalised linear mixed models. The qualitative methods used included constant comparative analysis, framework analysis, thematic analysis and content analysis. Hypothesis testing alone was used by two (18%) of the studies to gain estimates of the effectiveness of the intervention. Content analysis was the method of choice for the one study which only used qualitative methods. Two studies (18%) did not specify the method of analysis that was used.

### Remaining feasibility concerns and modifications required

Some feasibility concerns remained for the completed studies. One study observed differential recruitment success due to possible response bias, yet still deemed the main trial to be feasible without changes [22]. Two studies also concluded their studies were feasible without changes, despite some remaining concerns.

Even for those studies that listed no remaining concerns, changes were still intended to be made. These included changes to: the intervention [23], study procedures [23, 24] and data collection methods [24]. One study which identified several barriers, found issues predominately relating to time and resource availability [25]. The study was found to be feasible with several modifications, including the introduction of an adherence and retention package. One of the studies did not specify whether the main trial would be going ahead as a result of the feasibility study [26].

The majority of the studies did not specify whether the participants (55%) or clusters (64%) from the feasibility study would be taking part in the future trial. For four of the studies (36%) the commitment to using a SW-CRT design for the future definitive trial was not as strong as for the other trials. For these trials the stepped-wedge design was considered to be the most appropriate design for a future trial or the feasibility study itself was an SW-CRT. The only study to specifically assess the feasibility of using the stepped-wedge trial design, was one of the feasibility studies that was itself of a stepped-wedge design [21]. This feasibility study is described in [Additional File 1] along with a critical appraisal of its objectives.

## Discussion

Through a systematic review of the published literature we have identified 11 feasibility studies (eight reports, three protocols) conducted to inform SW-CRTs. Given the increasing frequency with which SW-CRTs are being used, it would be expected that there would be a greater number of feasibility studies published for these trials. This would suggest that few feasibility studies are being conducted in advance of running a definitive SW-CRT or that conducted feasibility studies are not being published. Furthermore, of the few SW-CRTs that have published feasibility studies, few have assessed the feasibility issues surrounding the use of the SW-CRT design itself. Given the complexities of the trial design, especially around the timings of the roll-out of the intervention under evaluation, evaluations using this trial design will be at risk of not delivering on their objectives.

The SW-CRT is an emerging, innovative and potentially very useful yet complex study design [3, 5, 6]. It has been shown to be particularly useful for the evaluation of interventions that would have been rolled out regardless of the trial taking place, as the implementation can often proceed in much the same way whilst providing randomised evidence of effectiveness [3]. In this way SW-CRTs are assisting in the move towards more pragmatic trials to answer routine pragmatic healthcare questions. There have been many reviews of SW-CRTs [1, 4, 16, 17, 27–32]. However, most reviews have focussed on statistical methodology (particularly sample size) and quality of reporting and none have looked at the use of feasibility studies for these trials. When designing a SW-CRT, there will often be aspects of the design that cannot be informed by previous trials, systematic reviews, routine data etc. and this information might only be gained through the use of a feasibility study. Obtaining this additional information can improve the feasibility of the designed trial.

We ascertained the design characteristics and rationale of the identified studies, in order to see how feasibility studies are currently being used to inform the design of SW-CRTs. In addition, we ascertained the processes employed by these studies for determining progression to a main trial, in order to see how feasibility studies are being used to inform SW-CRTs.

The studies varied considerably in both their size and duration, with some studies being completed in 16 weeks whilst others took two years and some studies requiring 16 participants whilst others included observations from more than 26,000 individuals. Many of the sample sizes lacked clear justification. Three studies did not provide any rationale for the size of the study and so it is not possible to determine whether the studies were large enough to accomplish their objectives or whether they might be excessively large. Under the CONSORT 2010 statement extension for randomised pilot and feasibility trials [10] the rationale for the numbers included in the study should be provided, especially for those studies where estimation of parameters such as recruitment rates is an objective. For many of the studies one of the main aims was to estimate the potential effectiveness of the intervention, which should not feature as an objective of a feasibility study as it will not be sufficiently powered for this [12]. Therefore, the decision as to whether to continue with the study or to progress to the definitive trial should not be based on any estimate of potential effectiveness from the feasibility study.

Only one of the feasibility studies aimed to assess the feasibility of using the stepped-wedge design, despite three of the studies being of a stepped-wedge design themselves. One study investigated the time taken to complete the study procedures, whereas the rest of the studies were mostly assessing the feasibility and acceptability of the intervention itself. With the complexity of the design of a SW-CRT, it is surprising to see so few of the studies investigating issues that are specific to the SW-CRT design. However, with the current dearth of papers describing the practical challenges of conducting a trial of this design, maybe this comes as less of a surprise. A small number of stepped-wedge trials that have been published, have reported the challenges faced [33]. Known challenges include delays in the start of the trial, poor recruitment and limited quantity and quality of data [27, 33, 34]. Many of which could be investigated using a feasibility study. Less than half of the identified studies were registered. The importance of registering feasibility studies has been highlighted by the CONSORT 2010 statement extension for randomised pilot and feasibility trials [10] and we reiterate this point. By registering feasibility studies it can make them easier to identify. Once identified these studies can be used to help inform the design of future SW-CRTs, by highlighting identified feasibility issues associated with this design.

### Strengths and limitations

Our review used a pre-specified search strategy, inclusion and exclusion criteria and duplicate data extraction in order to minimise potential sources of bias. The search strategy included many terms used for SW-CRTs, based on those included in other reviews [1, 16, 17] in an attempt to capture those studies using some of the less common terms. Yet, despite our best efforts there is still the potential for some selection bias as our search will not have captured those studies using other terms to describe the stepped-wedge design and non-English language studies. In addition, the results of our search will be limited to feasibility studies that specify that the main trial will be a SW-CRT in the title or abstract. Another added complexity and potential limitation is that feasibility studies often go unpublished [7, 12]. A recent review of feasibility studies funded by the National Institute for Health Research’s (NIHR) Research for Patient Benefit (RfPB) programme found almost half of the studies that they looked at had not published results [35]. We included studies self-defining as either pilot or feasibility studies; but other studies may have used other self-defining terminology to describe these studies - although this is likely to improve with the publication of the CONSORT 2010 statement extension for randomised pilot and feasibility trials [10].

Further work is required to highlight to researchers all of the potential feasibility issues associated with the SW-CRT, how some issues become more serious when using the stepped-wedge design and to promote the use of feasibility studies to inform these trials. The work presented here is part of a larger programme funded by the National Institute for Health Research (NIHR), which intends to identify the feasibility issues encountered by SW-CRTs and ultimately lead to the development of guidance on how feasibility studies can be conducted for SW-CRTs.

## Conclusions

Published feasibility studies to inform SW-CRTs are scarce and those that are being published do not aim to investigate many of the issues specific to this design of trial. SW-CRTs are complex and compared to other designs they are relatively inflexible to change once the trial has commenced. There is the potential for feasibility studies to be really informative in the designing of SW-CRTs, improving their design and giving them a greater chance of being completed successfully, on time and with the required sample size. We highlight the importance of conducting a feasibility study prior to any SW-CRT and encourage the publication of the findings in order to help other researchers planning on conducting a SW-CRT. We also encourage the published reports of completed SW-CRTs to highlight the challenges faced during the trial in order to help future trials to avoid encountering the same issues and provide them with the opportunity to investigate solutions to these issues during their own feasibility studies.

## Additional files

Additional file 1: Case study. Critical appraisal of the feasibility study published by Chari et al. [21] (DOCX 18 kb)

Additional file 2: List of included studies. The reference list of the 11 feasibility studies included in this review. (DOCX 14 kb)

Additional file 3: Characteristics of identified feasibility studies by study. The information on study characteristics summarised in Table 2, provided by study. (DOCX 15 kb)

Additional file 4: Rationales given for conducting the identified feasibility studies by study. The information on rationales given for conducting the feasibility study summarised in Table 3, provided by study. (DOCX 16 kb)

## References

[1] Mdege ND, Man M, Taylor (nee Brown), Celia A, Torgerson DJ: systematic review of stepped wedge cluster randomized trials shows that design is particularly used to evaluate interventions during routine implementation**.** J Clin Epidemiol 2011, 64(9):936–948. 10.1016/j.jclinepi.2010.12.003 21411284

[2] The Gambia Hepatitis Intervention Study (1987). The Gambia hepatitis study group. Cancer Res.

[3] Hemming K, Haines TP, Chilton PJ, Girling AJ, Lilford RJ (2015). The stepped wedge cluster randomised trial: rationale, design, analysis, and reporting. BMJ (Online).

[4] Grayling MJ, Wason JM, Mander AP (2017). Stepped wedge cluster randomized controlled trial designs: a review of reporting quality and design features. Trials.

[5] Kotz D, Spigt M, Arts ICW, Crutzen R, Viechtbauer W (2012). Use of the stepped wedge design cannot be recommended: a critical appraisal and comparison with the classic cluster randomized controlled trial design. J Clin Epidemiol.

[6] Zhan Z, Van Den Heuvel ER, Doornbos PM, Burger H, Verberne CJ, Wiggers T, De Bock GH (2014). Strengths and weaknesses of a stepped wedge cluster randomized design: its application in a colorectal cancer follow-up study. J Clin Epidemiol.

[7] Arain M, Campbell MJ, Cooper CL, Lancaster GA (2010). What is a pilot or feasibility study? A review of current practice and editorial policy. BMC Med Res Methodol.

[8] Whitehead AL, Sully BGO, Campbell MJ (2014). Pilot and feasibility studies: is there a difference from each other and from a randomised controlled trial?. Contemporary Clinical Trials.

[9] Eldridge S, Kerry S (2012). A practical guide to cluster randomised trials in health services research.

[10] Eldridge SM, Chan CL, Campbell MJ, Bond CM, Hopewell S, Thabane L, Lancaster GA (2016). CONSORT 2010 statement: extension to randomised pilot and feasibility trials. Pilot and Feasibility Studies.

[11] Thabane L, Ma J, Chu R, Cheng J, Ismaila A, Rios LP, Robson R, Thabane M, Giangregorio L, Goldsmith CH (2010). A tutorial on pilot studies: the what, why and how. BMC Med Res Methodol.

[12] Lancaster GA, Dodd S, Williamson PR (2004). Design and analysis of pilot studies: recommendations for good practice. J Eval Clin Pract.

[13] Eldridge SM, Lancaster GA, Campbell MJ, Thabane L, Hopewell S, Coleman CL, Bond CM (2016). Defining feasibility and pilot studies in preparation for randomised controlled trials: development of a conceptual framework. PLoS One.

[14] Caille A, Kerry S, Tavernier E, Leyrat C, Eldridge S, Giraudeau B (2016). Timeline cluster: a graphical tool to identify risk of bias in cluster randomised trials. BMJ.

[15] Kristunas CA, Hemming K, Eborall HC, Gray LJ: The use of feasibility studies for stepped-wedge cluster randomised trials: protocol for a review of impact and scope**.** BMJ Open 2017, 7(e017290). 10.1136/bmjopen-2017-017290 PMC5642661 28765139

[16] Brown CA, Lilford RJ (2006). The stepped wedge trial design: a systematic review. BMC Med Res Methodol.

[17] Beard E, Lewis JJ, Copas A, Davey C, Osrin D, Baio G, Thompson JA, Fielding KL, Omar RZ, Ononge S, Hargreaves J, Prost A (2015). Stepped wedge randomised controlled trials: systematic review of studies published between 2010 and 2014. Trials.

[18] Moher D, Shamseer L, Clarke M, Ghersi D, Liberati A, Petticrew M, Shekelle P, Stewart L, PRISMA-P Group: Preferred reporting items for systematic review and meta-analysis protocols (PRISMA-P) 2015 statement**.** Systematic Reviews 2015, 4(1):1. 10.1186/2046-4053-4-1 PMC4320440 25554246

[19] Brady MC, Stott D, Weir CJ, Chalmers C, Sweeney P, Donaldson C, Barr J, Barr M, Pollock A, Mcgowan S, Bowers N, Langhorne P (2015). Clinical and cost effectiveness of enhanced oral healthcare in stroke care settings (SOCLE II): a pilot, stepped wedge, cluster randomized, controlled trial protocol. Int J Stroke.

[20] Carrico AW, Nil E, Sophal C, Stein E, Sokunny M, Yuthea N, Evans JL, Ngak S, Maher L, Page K (2016). Behavioral interventions for Cambodian female entertainment and sex workers who use amphetamine-type stimulants. J Behav Med.

[21] Chari SR, Smith S, Mudge A, Black AA, Figueiro M, Ahmed M, Devitt M, Haines TP (2016). Feasibility of a stepped wedge cluster RCT and concurrent observational sub-study to evaluate the effects of modified ward night lighting on inpatient fall rates and sleep quality: a protocol for a pilot trial. Pilot feasibility stud.

[22] Becker SJ, Squires DD, Strong DR, Barnett NP, Monti PM, Petry NM (2016). Training opioid addiction treatment providers to adopt contingency management: a prospective pilot trial of a comprehensive implementation science approach. Subst Abus.

[23] Ettema R, Schuurmans MJ, Schutijser B, Van Baar M, Kamphof N, Kalkman CJ (2015). Feasibility of a nursing intervention to prepare frail older patients for cardiac surgery: a mixed-methods study. Eur J Cardiovasc Nurs.

[24] McIlvennan CK, Thompson JS, Matlock DD, Cleveland JC, Dunlay SM, Larue SJ, Lewis EF, Patel CB, Walsh MN, Allen LA (2016). A multicenter trial of a shared decision support intervention for patients and their caregivers offered destination therapy for advanced heart failure: DECIDE-LVAD. J Cardiovasc Nurs.

[25] Napua M, Pfeiffer JT, Chale F, Hoek R, Manuel J, Michel C, Cowan JG, Cowan JF, Gimbel S, Sherr K, Gloyd S, Chapman RR, Option B (2016). + in Mozambique: formative research findings for the Design of a Facility-Level Clustered Randomized Controlled Trial to improve ART retention in antenatal care. J Acquir Immune Defic Syndr.

[26] Tume L (2005). A 3-year review of emergency PICU admissions from the ward in a specialist cardio-respiratory Centre. Care of the Critically Ill.

[27] Prost A, Binik A, Abubakar I, Roy A, De Allegri M, Mouchoux C, Dreischulte T, Ayles H, Lewis JJ, Osrin D. Logistic, ethical, and political dimensions of stepped wedge trials: critical review and case studies. Trials. 2015;16(1). 10.1186/s13063-015-0837-4 PMC4538739 26278521

[28] Baio G, Copas A, Ambler G, Hargreaves J, Beard E, Omar R (2015). Sample size calculation for a stepped wedge trial. Trials.

[29] Martin J, Taljaard M, Girling A, Hemming K (2016). Systematic review finds major deficiencies in sample size methodology and reporting for stepped-wedge cluster randomised trials. BMJ Open.

[30] Barker D, McElduff P, D'Este C, Campbell MJ (2016). Stepped wedge cluster randomised trials: a review of the statistical methodology used and available. BMC Med Res Methodol.

[31] Kristunas C, Morris T, Gray L (2017). Unequal cluster sizes in stepped-wedge cluster randomised trials: a systematic review. BMJ Open.

[32] Taljaard M, Hemming K, Shah L, Giraudeau B, Grimshaw JM, Weijer C (2017). Inadequacy of ethical conduct and reporting of stepped wedge cluster randomized trials: results from a systematic review. Clinical Trials.

[33] Heim N, van Stel HF, Ettema RG, van der Mast RC, Inouye SK, Schuurmans MJ (2017). HELP! Problems in executing a pragmatic, randomized, stepped wedge trial on the hospital elder life program to prevent delirium in older patients. Trials.

[34] Hargreaves JR, Copas AJ, Beard E, Osrin D, Lewis JJ, Davey C, Thompson JA, Baio G, Fielding KL, Prost A. Five questions to consider before conducting a stepped wedge trial. Trials. 2015;16(1). 10.1186/s13063-015-0841-8 PMC4538743 26279013

[35] Morgan B, Hejdenberg J, Hinrichs-Krapels S, Armstrong D (2018). Do feasibility studies contribute to, or avoid, waste in research?. PLoS One.

