# Supplementary material for: The current use of feasibility studies in the assessment of feasibility for stepped-wedge cluster randomised trials: a systematic review
Source: BMC Med Res Methodol. 2019 Jan 10;19:12. doi: 10.1186/s12874-019-0658-3 (PMC6327386; doi:10.1186/s12874-019-0658-3)
Supplement: Supplementary file 1 — Case study. Critical appraisal of the feasibility study published by Chari et al. [21] (DOCX 18 kb) [file 12874_2019_658_MOESM1_ESM.docx]

Chari et al. describe the protocol for a feasibility study of a stepped-wedge cluster randomised trial (SW-CRT) to evaluate the effects of modified ward night lighting on inpatient fall rates and sleep quality. The intervention consisted of the installation of LED strip lighting around the exterior door frames of the bathroom, above the washbasin and behind and adjacent to the toilet. To confirm the feasibility of the trial it was deemed necessary to conduct a feasibility study with a stepped wedge design, in order to assess the “potential for a variety of operational and logistic challenges in installing new equipment over entire wards”. These are good objectives for a feasibility study for a SW-CRT, as the staggered implementation of the intervention can complicate the logistics of the trial.

Due to funding constraints, the feasibility study was restricted to six inpatient wards in one site and a data collection period of 14 months. Implementation of the intervention in six wards was deemed to provide sufficient opportunities to observe all practical challenges that may be encountered and provide adequate time to test and modify the implementation approach. The primary objective was to understand the feasibility of the SW-CRT design; other objectives were to test a range of proposed data collection methods; inform sample size calculations; confirm intervention features; and finalise the cost-effectiveness modelling approach.

Besides these predominately process type motivations, the study also set scientific type objectives of assessing the directionality of the intervention effect and the proportion of patients having one or more falls as an inpatient; including a stopping rule based on the main clinical outcome (rate of falls). These are not considered appropriate objectives for a feasibility study, since the study will not be sufficiently powered to answer these questions. A stopping rule should instead be based around the boundaries of the confidence interval rather than the point estimate.

A concurrent observational sub-study was also conducted. One patient per ward per month were to be recruited and detailed patient-level outcomes measured through a wrist actigraph, environmental sensors and self-reported measures provided during interviews. The collection of this data was intended to help understand the “potential mechanisms of effect for the intervention” and the environmental sensors were intended to assess the fidelity of the intervention. On completion of the intervention implementation, face-to-face interviews were to be held with ward staff to gauge satisfaction with the intervention and identify ways to improve the implementation approach and intervention characteristics. These are all valuable things to investigate during a feasibility study.

Chari et al. conclude that the primary value of undertaking this feasibility study is in confirming whether the approach is suitable and in generating sufficient pilot data to “test the postulated mechanisms of effect, to confirm the feasibility and utility of proposed data collection methods and to finalise intervention features in advance of a larger follow-up trial”.
