# Supplementary material for: The current use of feasibility studies in the assessment of feasibility for stepped-wedge cluster randomised trials: a systematic review
Source: BMC Med Res Methodol. 2019 Jan 10;19:12. doi: 10.1186/s12874-019-0658-3 (PMC6327386; doi:10.1186/s12874-019-0658-3)
Supplement: Supplementary file 2 — List of included studies. The reference list of the 11 feasibility studies included in this review. (DOCX 14 kb) [file 12874_2019_658_MOESM2_ESM.docx]

Becker SJ, Squires DD, Strong DR, Barnett NP, Monti PM, Petry NM. Training opioid addiction treatment providers to adopt contingency management: a prospective pilot trial of a comprehensive implementation science approach. Substance abuse. 2016 Jan 2;37(1):134-40.

Brady MC, Stott DJ, Norrie J, Chalmers C, St George B, Sweeney PM, Langhorne P. Developing and evaluating the implementation of a complex intervention: using mixed methods to inform the design of a randomised controlled trial of an oral healthcare intervention after stroke. Trials. 2011 Jul 5;12(1):168.

Brady MC, Stott D, Weir CJ, Chalmers C, Sweeney P, Donaldson C, Barr J, Barr M, Pollock A, McGowan S, Bowers N. Clinical and cost effectiveness of enhanced oral healthcare in stroke care settings (SOCLE II): A pilot, stepped wedge, cluster randomized, controlled trial protocol. International Journal of Stroke. 2015 Aug;10(6):979-84.

Carrico AW, Nil E, Sophal C, Stein E, Sokunny M, Yuthea N, Evans JL, Ngak S, Maher L, Page K. Behavioral interventions for Cambodian female entertainment and sex workers who use amphetamine-type stimulants. Journal of behavioral medicine. 2016 Jun 1;39(3):502-10.

Chari SR, Smith S, Mudge A, Black AA, Figueiro M, Ahmed M, Devitt M, Haines TP. Feasibility of a stepped wedge cluster RCT and concurrent observational sub-study to evaluate the effects of modified ward night lighting on inpatient fall rates and sleep quality: a protocol for a pilot trial. Pilot and feasibility studies. 2016 Jan 7;2(1):1.

Escobar GJ, Turk BJ, Ragins A, Ha J, Hoberman B, LeVine SM, Ballesca MA, Liu V, Kipnis P. Piloting electronic medical record–based early detection of inpatient deterioration in community hospitals. Journal of hospital medicine. 2016 Nov 1;11(S1).

Ettema R, Schuurmans MJ, Schutijser B, van Baar M, Kamphof N, Kalkman CJ. Feasibility of a nursing intervention to prepare frail older patients for cardiac surgery: A mixed-methods study. European Journal of Cardiovascular Nursing. 2015 Aug;14(4):342-51.

Feng R, Li K, Cheng J, Xie S, Chai J, Wei P, Wang D. Toward integrated and sustainable prevention against diabetes in rural China: study rationale and protocol of eCROPS. BMC endocrine disorders. 2013 Aug 7;13(1):28.

McIlvennan CK, Thompson JS, Matlock DD, Cleveland Jr JC, Dunlay SM, LaRue SJ, Lewis EF, Patel CB, Walsh MN, Allen LA. A multicenter trial of a shared decision support intervention for patients and their caregivers offered destination therapy for advanced heart failure: DECIDE-LVAD: rationale, design, and pilot data. Journal of Cardiovascular Nursing. 2016 Nov 1;31(6):E8-20.

Napúa M, Pfeiffer JT, Chale F, Hoek R, Manuel J, Michel C, Cowan JG, Cowan JF, Gimbel S, Sherr K, Gloyd S. Option B+ in Mozambique: formative research findings for the design of a facility-level clustered randomized controlled trial to improve ART retention in antenatal care. Journal of acquired immune deficiency syndromes (1999). 2016 Aug 1;72(Suppl 2):S181.

Tume LN, Preston J, Blackwood B. Parents' and young people's involvement in designing a trial of ventilator weaning. Nursing in critical care. 2016 May 1;21(3).
