# Supplementary material for: The current use of feasibility studies in the assessment of feasibility for stepped-wedge cluster randomised trials: a systematic review
Source: BMC Med Res Methodol. 2019 Jan 10;19:12. doi: 10.1186/s12874-019-0658-3 (PMC6327386; doi:10.1186/s12874-019-0658-3)
Supplement: Supplementary file 3 — Characteristics of identified feasibility studies by study. The information on study characteristics summarised in Table 2, provided by study. (DOCX 15 kb) [file 12874_2019_658_MOESM3_ESM.docx]

| **Lead author (year)** | **Becker (2016)** | **Brady (2011)** | **Brady (2015)** | **Carrico (2016)** | **Chari (2016)** | **Escobar (2016)** | **Ettema (2015)** | **Feng (2013)** | **McIlvennan (2016)** | **Napúa (2016)** | **Tume (2016)** |
| --- | --- | --- | --- | --- | --- | --- | --- | --- | --- | --- | --- |
| **Study registered** | Not specified | Not specified | Yes | Yes | Yes | Not specified | Not specified | Yes | Yes | Not specified | Not specified |
| **Report of findings or protocol** | Report | Report | Protocol | Report | Protocol | Report | Report | Protocol | Report | Report | Report |
| **Study described as** | Pilot | Pilot | Pilot | Not described | Pilot | Pilot | Feasibility | Pilot | “Acceptability and feasibility pilot study” | Formative research | Consultation exercise |
| **Internal or external feasibility study** | External | External | External | Internal | External | Unclear | External | Internal | External | External | External |
| **Type of research** | Quantitative | Mixed-methods | Mixed-methods | Mixed-methods | Quantitative | Quantitative | Mixed-methods | Unclear | Mixed-methods | Mixed-methods | Qualitative |
| **Quantitative design** | Parallel | Single-arm | Stepped-wedge | Stepped-wedge | Stepped-wedge | Single-arm | Single-arm | Unclear | Single-arm | Observational | Qualitative |
| **Randomised** | No | No | Yes | Yes | Yes | No | No | No | No | No | No |
| **Setting** | Healthcare | Healthcare | Healthcare | Community | Healthcare | Healthcare | Healthcare | Community | Healthcare | Healthcare | Healthcare |
| **Cluster type** | Clinics | Wards | Wards | Geographical areas | Wards | Hospitals | Hospitals | Geographical areas | Hospitals | Clinics | Wards |
| **Study duration (months)** | 12 | 4.4 | 16 | 3.7 | 14 | 104 | Not specified | Unclear | 5 | 12 | 6 |
| **Number of clusters** | 15 | 1 | 4 | 6 | 6 | 2 | 3 | 3 | 1 | 6 | NA |
| **Participant type** | Healthcare professionals | Healthcare professionals and patients | Healthcare professionals and patients | Female entertainment and sex workers | Patients | Patients | Patients | Healthcare professionals and patients | Patients | Healthcare professionals and patients | Patients |
| **Number of participants** | 60 | 40 | 460 | 138 | 7500 | 26386 | 79 | Not given | 19 | 154 | 16 |
| **Rationale for sample size** | Not stated | Convenience | Convenience | Convenience | Based on main clinical outcome | Not stated | Based on main clinical outcome | Convenience | Convenience | Not stated | Convenience |
