# Supplementary material for: The current use of feasibility studies in the assessment of feasibility for stepped-wedge cluster randomised trials: a systematic review
Source: BMC Med Res Methodol. 2019 Jan 10;19:12. doi: 10.1186/s12874-019-0658-3 (PMC6327386; doi:10.1186/s12874-019-0658-3)
Supplement: Supplementary file 4 — Rationales given for conducting the identified feasibility studies by study. The information on rationales given for conducting the feasibility study summarised in Table 3, provided by study. (DOCX 16 kb) [file 12874_2019_658_MOESM4_ESM.docx]

| **Lead author (year)** | **Becker (2016)** | **Brady (2011)** | **Brady (2015)** | **Carrico (2016)** | **Chari (2016)** | **Escobar (2016)** | **Ettema (2015)** | **Feng (2013)** | **McIlvennan (2016)** | **Napúa (2016)** | **Tume (2016)** |
| --- | --- | --- | --- | --- | --- | --- | --- | --- | --- | --- | --- |
| **Process type motivations** |  |  |  |  |  |  |  |  |  |  |  |
| Acceptability of intervention |  | ✓ | ✓ | ✓ | ✓ | ✓ | ✓ |  | ✓ |  |  |
| Identify issues/barriers to implementation |  |  | ✓ | ✓ | ✓ | ✓ | ✓ |  |  | ✓ |  |
| Adherence to intervention |  | ✓ | ✓ | ✓ |  |  | ✓ |  |  |  |  |
| Development of intervention |  |  |  |  |  | ✓ |  | ✓ |  | ✓ |  |
| Retention rate estimation |  |  |  | ✓ |  |  | ✓ |  | ✓ |  |  |
| Determining outcome measures |  | ✓ |  |  |  |  |  |  |  |  | ✓ |
| Test data collection methods |  |  | ✓ |  | ✓ |  |  |  |  |  |  |
| Assess amount of missing data | ✓ |  |  |  |  |  |  |  | ✓ |  |  |
| Participant satisfaction |  |  |  |  |  |  |  |  | ✓ |  |  |
| Assess values-treatment concordance |  |  |  |  |  |  |  |  | ✓ |  |  |
| Acceptability relevance and importance of trial |  |  |  |  |  |  |  |  |  |  | ✓ |
| Test sampling methodologies |  |  | ✓ |  |  |  |  |  |  |  |  |
| Test feasibility of using stepped-wedge design |  |  |  |  | ✓ |  |  |  |  |  |  |
| **Resource type motivations** |  |  |  |  |  |  |  |  |  |  |  |
| Resources used in intervention |  |  | ✓ |  |  |  |  |  |  |  |  |
| Post-intervention impact on service use and staff time |  |  | ✓ |  |  |  |  |  |  |  |  |
| Time taken to complete study procedures |  |  |  |  |  |  |  |  | ✓ |  |  |
| Waiting and consultation times |  |  |  |  |  |  |  |  |  | ✓ |  |
| Patient volumes and staffing levels during study |  |  |  |  |  |  |  |  |  | ✓ |  |
| **Management type motivations** |  |  |  |  |  |  |  |  |  |  |  |
| None reported | ✓ | ✓ | ✓ | ✓ | ✓ | ✓ | ✓ | ✓ | ✓ | ✓ | ✓ |
| **Scientific type motivations** |  |  |  |  |  |  |  |  |  |  |  |
| Potential effectiveness of intervention | ✓ | ✓ | ✓ |  | ✓ |  |  |  | ✓ |  |  |
| Assess cost-effectiveness/theoretical cost saving |  |  | ✓ |  | ✓ |  | ✓ |  |  |  |  |
| Inform sample size calculation |  | ✓ | ✓ |  | ✓ |  |  |  |  |  |  |
| Assess intervention safety |  |  | ✓ |  | ✓ |  |  |  |  |  |  |
| Estimate Intra Cluster Correlation Coefficient (ICC) |  |  | ✓ |  |  |  |  |  |  |  |  |
| Assess correlation between measures | ✓ |  |  |  |  |  |  |  |  |  |  |
| Assess distributional properties of measures | ✓ |  |  |  |  |  |  |  |  |  |  |
